# Photovoltaic performance of MOF-derived transition metal doped titania-based photoanodes for DSSCs

**DOI:** 10.1038/s41598-023-33565-6

**Published:** 2023-04-18

**Authors:** C. Nizamudeen, R. Krishnapriya, M. S. Mozumder, A-H. I. Mourad, T. Ramachandran

**Affiliations:** 1 Mechanical and Aerospace Engineering Department, College of Engineering, United Arab Emirate University, 15551 Al Ain, United Arab Emirates; 2 Department of Chemistry, Indian Institute of Technology Jodhpur, Jodhpur, 342037 Rajasthan India; 3 Department of Chemical and Petroleum Engineering, College of Engineering, United Arab Emirate University, 15551 Al Ain, United Arab Emirates; 4 National Water and Energy Centre, United Arab Emirate University, 15551 Al Ain, United Arab Emirates; 5 On Leave from Mechanical Design Department, Faculty of Engineering, Helwan University, Cairo, Egypt

**Keywords:** Materials for devices, Materials for energy and catalysis, Nanoscale materials

## Abstract

The enduring effort toward stabilizing and improving the efficiency of dye-sensitized solar cells (DSSCs) has stirred the solar research community to follow innovative approaches. Current research centered on electrode materials design, which improves photoanodes' light-harvesting efficiency (LHE). Metal–Organic Frameworks (MOFs) are a new family of materials that can be used as competent materials due to their desirable qualities, including high porosity, flexible synthesis methodology, high thermal and chemical stability, and good light-harvesting capabilities. MOF-derived porous photoanodes can effectively adsorb dye molecules and improve LHE, resulting in high power conversion efficiency (PCE). Doping is a prospective methodology to tune the bandgap and broaden spectral absorption. Hence, a novel and cost-effective synthesis of high surface area transition metal (TM) doped TiO_2_ nanocrystals (NCs) via the metal–organic framework route for DSSCs is reported here. Among the TM dopants (i.e., Mn, Fe, Ni), a remarkable PCE of 7.03% was obtained for nickel-doped samples with increased Jsc (14.66 mA/cm^2^) due to the bandgap narrowing and porous morphology of TiO_2_. The findings were further confirmed using electrochemical impedance spectroscopy (EIS) and dye-desorption experiments. The present study expedites a promising way to enhance the LHE for many innovative optoelectronic devices.

## Introduction

As incipient photovoltaic technology, dye-sensitized solar cells (DSSCs) have been conscientiously and widely scrutinized with a marked upward trend in their efficiency over the last few years^1–6^. The optimum performance of a DSSC device predominantly banks on the nanostructured electron transporting layer of titanium dioxide (TiO_2_). TiO_2_ is the most expansively inspected semiconductor material for DSSC, owing to its chemically and thermally stable nature, cost-effectiveness, non-toxicity, and facile applicability^7–14^. The application potential of TiO_2_ is confined due to the wide bandgap (3.2 eV), which limits the light absorption only in the UV light region, the high recombination rate of photo-injected electrons, the tendency to agglomerate, and low quantum efficiency^15^. Extensive research was conducted to improve the power conversion efficiency (PCE) of DSSC by carefully tailoring the morphology to make the most of the dye-TiO_2_ interface, consequently enhancing light absorption and electron transport^16^. Subsequently, altering the characteristic electronic properties of TiO_2_ is getting huge scientific considerations worldwide^17–21^. An effective way of modifying the electronic properties of TiO_2_ is doping, i.e., the intentional incorporation of impurity atoms into the TiO_2_ crystal lattice. In photovoltaic (PV) applications, this methodology (i.e., doping) can increase the spectral response and stimulate effective charge separation^22^.

For successful doping, the atomic radius of the dopant should match that of the ion it replaces to avoid lattice distortion, which generates defects that typically affect DSSC performance. In the case of cation doping, the change of Ti with a new cation influences the dye adsorption due to different binding strengths amongst the dye molecule and the dopant ions. Also, the introduction of dopants during the TiO_2_ synthesis hinders the crystal growth mechanism, causing the formation of nanocrystals (NCs)^23,24^. Hierarchical TiO_2_ nanostructures formed from these NC assemblies can offer a larger surface area for dye-loading, leading to excellent light absorption and current densities^25^. It benefits from using very thin photoanode film with fewer recombination sites. Many research works exploiting the doping methodology are found in the literature where dopants are introduced into the TiO_2_ lattice using anodization, pulsed laser deposition, spray pyrolysis, atomic layer deposition, sol–gel, hydrothermal, solvothermal, microwave electrochemical, and electrospinning methods etc^26–30^. Alkali metals, metalloids, non-metals, and transitional metals (TMs) were successfully applied as dopants^31–35^. Among these, TMs offer a wide range of new energy levels close to the conduction band (CB) of TiO_2_ owing to the partially filled d-orbitals, resulting in the effective tuning of CB structure^36^. TM ions have multiple valences with vacant d-electron structure, which can accommodate more electrons and can introduce different impurity levels in the band gap of TiO_2_. Thus these crystals can and become a shallow trap of photogenerated electrons or holes, for decreasing the recombination of electron–hole pairs.

Fabrication of doped NCs with specific size, compositions, and internal structure is a significant challenge due to the difference in the reactivity of the dopant and the host precursor. Also, the chances of dopant exclusion from the host lattice (self-purification) are high^37^. Therefore, it is significant to develop efficient synthesis methodologies for fabricating the doped semiconductor NCs. Recently, metal–organic frameworks (MOFs), i.e., hybrid metal ions (metal clusters) and multi-dentate organic linkers, are getting considerable attention as templates or precursors to synthesize semiconductor NCs by high-temperature pyrolysis^38–40^. Since MOF possesses periodic crystalline structures and porosities, precise control of the size, shape, composition, and structure, along with the integration of diverse functionalities, can be achieved in a single step^41^. Accordingly, the obtained NCs fabricated through this route often exhibit high surface area, tunable pore structures, controllable size, and functionalities. Studies reported that while using the MIL-125 MOF as the precursor for fabricating the doped TiO_2_ NCs, the dopant cations were well dispersed into the MOF frameworks during the adsorption process with the advantage of maintaining the 3D morphological topographies^42–44^. Li et al. utilized ZIF-8 MOFs for the first time for DSSC, who coated a very thin layer (2 nm) of ZIF-8 film on TiO_2_ photoanode. The study revealed that MOFs are potentially favorable shell materials for enhancing the Voc of DSSC^45^. Later, Dou et al. introduced photoanode made of thermolysis of MIL-125(Ti) MOF^46^. The hierarchical porous anatase TiO2 photoanodes showed an efficiency of 7.20% compared to P25 photoanode with 6.4% efficiency. Our previous study used MIL-125(Ti) MOFs to fabricate Co^2+^ doped TiO_2_ photoanodes for DSSCs^47^. The Co^2+^ doping methodology resulted in enhanced light absorption in the visible region when employed as photoanode resulting in enhanced PCE of 6.86% with promising photocurrent density (Jsc) of 13.96 mA cm^−2^. Despite this, there is little study on MOF-derived TiO_2_ materials for DSSCs in the literature. Also, TM doped TiO_2_ synthesized through MOF is not well-explored for the DSSC application. This inspires us to continue our research in order to synthesize diverse TM doped TiO_2_ for solar cell applications using the MOF approach.

Thus, this manuscript reports the synthesis of pristine and Mn, Fe, and Ni-doped TiO_2_ NCs via the MIL-125 MOF route. A series of TM doped TiO_2_ NCs with 10, 30, and 50 wt% of dopants were synthesized and a systematic comparative study on the dopant-induced PV performance for DSSCs was carried out. To the best of our knowledge, no such study has been published. The achieved results showed that the dopant could improve the structural, optical, and electronic properties of TiO_2_ and thereby improve the PV performance.

## Results

MIL-125 was used to prepare the TiO_2_ NCs by controlled thermolysis under air. To synthesize TM doped TiO_2_ NCs, 10, 30 and 50 mg of each TM precursor were added to the reaction mixture and the obtained doped samples were named as 10-X-TiO_2_, 30-X-TiO_2_ and 50-X-TiO_2_, respectively (where X = Mn, Fe or Ni). The primary identification of the TM doping was done by the color change observed for samples as shown in Fig. S1. The energy dispersive specta (EDS) of the doped samples further confirmed the presence of the metals Mn, Fe, Ni, Ti and O as shown in Fig. S2. The optical properties of the doped TiO_2_ were analyzed using UV visible spectroscopy. The bandgaps were measured using diffuse reflectance spectra following the theory of P. Kubelka and F. Munk and is given in Fig. S3^48^. The pristine TiO_2_ has the bandgap of 3.2 eV; however, after the metal doping at different concentrations, the bandgaps were reduced significantly and can be identified from Fig. S3. A schematic representation of band gap structure and possible crystal structure before and after doping are given in Figs. S4, S5 respectively.

**Figure 1:**
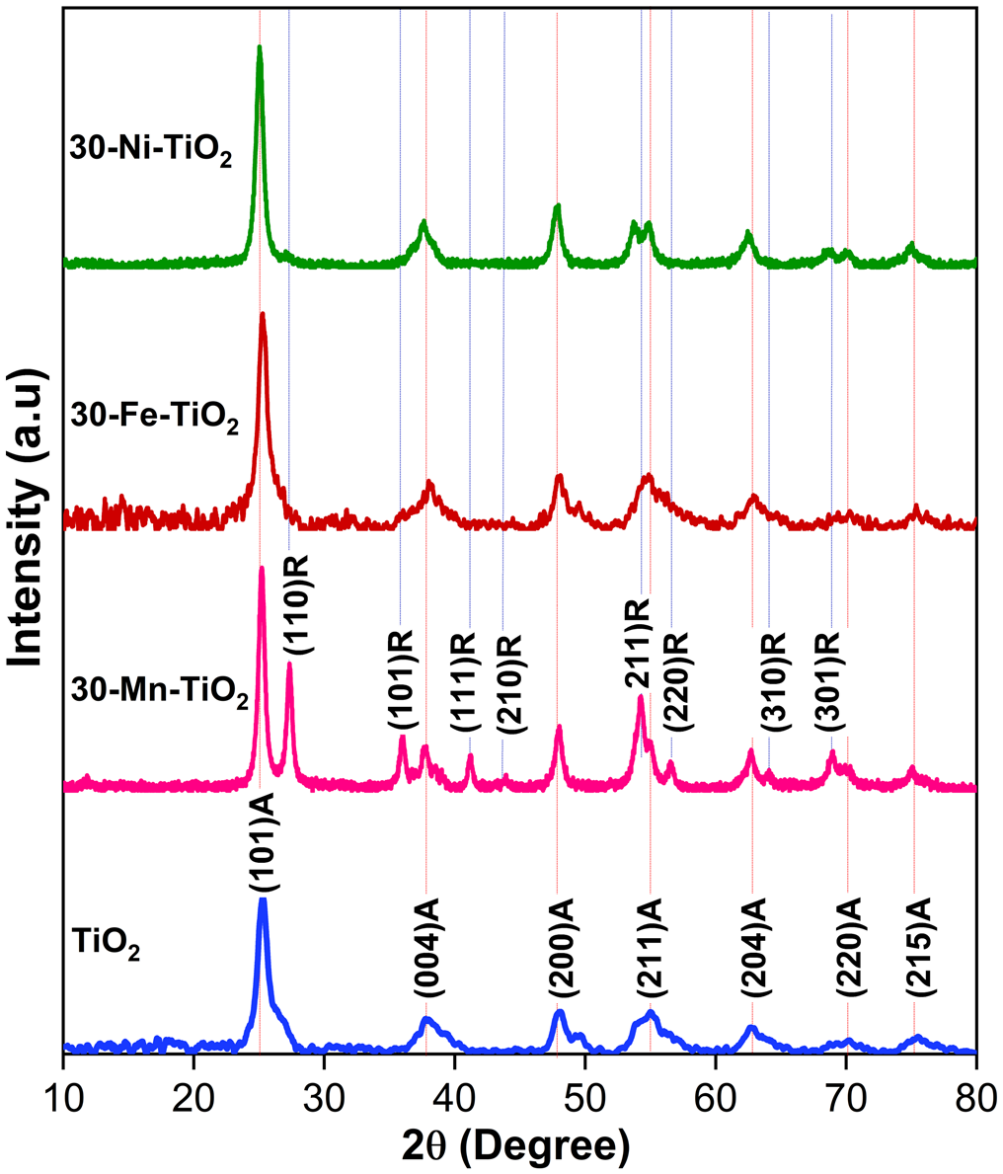
XRD pattern of calcined MIL-125 MOF derived TiO_2_ and 30 mg Mn, Fe and Ni doped TiO_2_ samples showing anatase and rutile phases.

**Figure 2:**
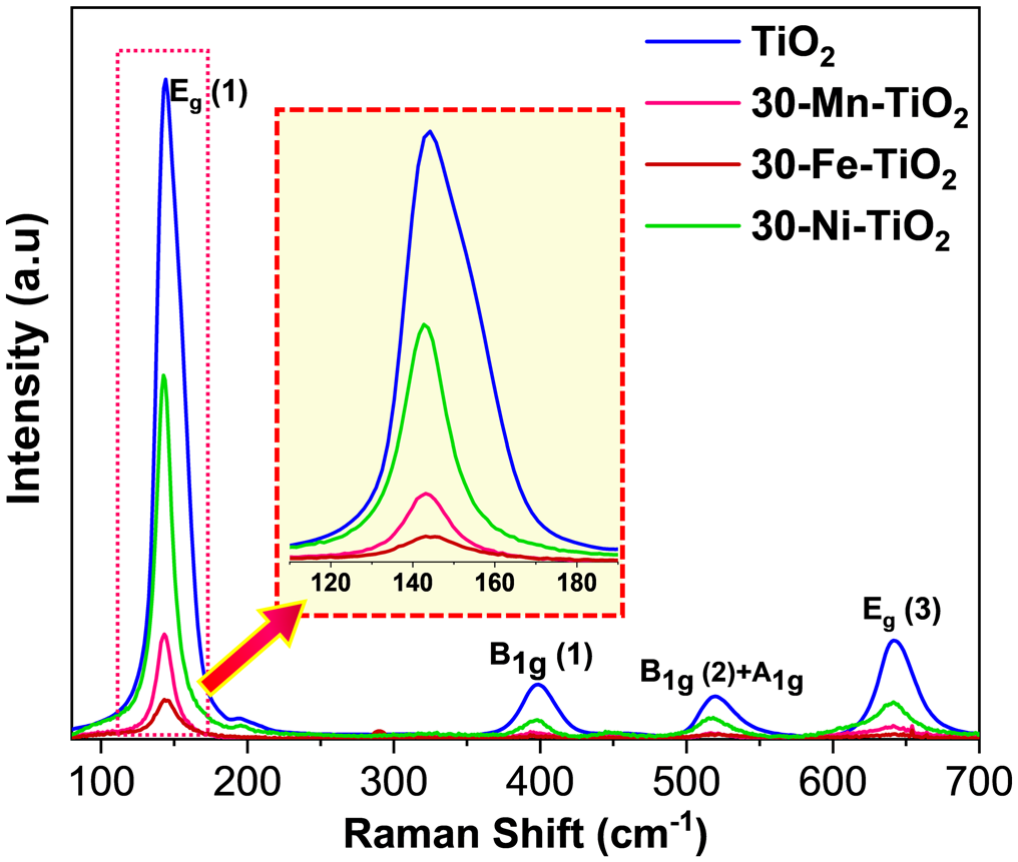
Raman spectra in the range of 100–700 cm^–1^ for the MIL-125 MOF derived TiO_2_ and 30 mg Mn, Fe and Ni doped TiO_2_ NCs showing the anatase phase. The inset figure showing the peak narrowing after the transition metal incorporation.

The phase identification and crystal structure of the samples were revealed by X-ray diffraction (XRD) studies. Figure 1 shows the XRD patterns of pristine and doped samples. The diffraction peaks of calcined undoped and 30 wt% metal-doped samples showed mainly anatase crystal structure. The pristine (undoped) sample showed peaks around 2Ɵ angles at 25.33, 37.78, 48.27, 55.02, 62.85, 70.14 and 75.46° which are indexed to (101), (004), (200), (211), (204), (220) and (215) of tetragonal crystal planes of pure anatase TiO_2_ according to the JCPDS card number 21–1272 49. However, the 30 wt% manganese doped TiO_2_ sample showed mixed phases of anatase and rutile. The peaks obtained at 2Ɵ values of 27.44, 35.96, 41.22, 43.75, 53.95, 56.59, 64.18, and 68.88 correspond to (110), (101), (111), (210), (211), (220), (310), and (301) tetragonal planes of rutile TiO_2_^50^. Iron and nickel doped TiO_2_ samples exhibited diffraction peaks for anatase phase. No other impurity peaks of any precursor or other oxide species were observed. The XRD peaks of all the doped samples exhibited intense sharp peaks. This is attributed to the increased crystallanity of the samples after metal doping. More insights on the local structure of TiO_2_ and the doping-induced structural changes can be understood deeply by Raman spectroscopy analysis. Figure 2 shows the Raman spectra of pristine and 30 wt% TM doped samples. All spectra illustrate the formation of anatase TiO_2_ crystal structure. As observed in XRD, the Raman spectra also confirm the absence of any impurity and secondary peaks. The sharp, intense peak obtained at 144 cm^−1^ corresponds to the characteristic Eg mode of anatase TiO_2_ that arises by symmetric stretching vibrations of O–Ti–O bonds. A low intensity (Eg) peak was observed at 197 cm^−1^. Apart from these, the peaks were obtained at 399, 518.9 and 643.74 cm^−1^ which correspond to B1g, A1g or B1g and Eg modes; in which B1g mode arises by symmetric bending and A1g mode by anti-symmetric bending vibrations of O–Ti–O bonds^51,52^. Any Raman modes are associated with stretching or bending vibration of Ti–O bonds. All the doped samples exhibited approximately the same bands of pristine TiO_2_ with lower intensity, and the results are consistent with XRD data. The shape and position of the Raman peaks are affected after doping and is attributed to the changes in anatase crystal structure, cleavage of vibrational phonon mode and oxygen vacancy^53,54^. No evident peaks related to the rutile phase were observed, confirming that all samples were mostly dominated by the anatase phase of TiO_2_. However, Mn doped TiO_2_ samples showed Raman peaks at 444 and 613 cm^−1^ corresponds to Eg and A1g modes of rutile TiO_2_. (Fig. S6). The scattering intensities of Raman modes were significantly decreased after doping with transition metals. It may be due to the breakdown of the lattice periodicity and long range translational crystal symmetry of TiO_2_ by the induced defects in the crystal lattice. Even though, the dopant concentration taken were same, the amount of dopant ions in the crystal structure will vary depending on the ionic size. This might be the possible reason for the intensity difference in the obtained Raman spectra.

**Figure 3:**
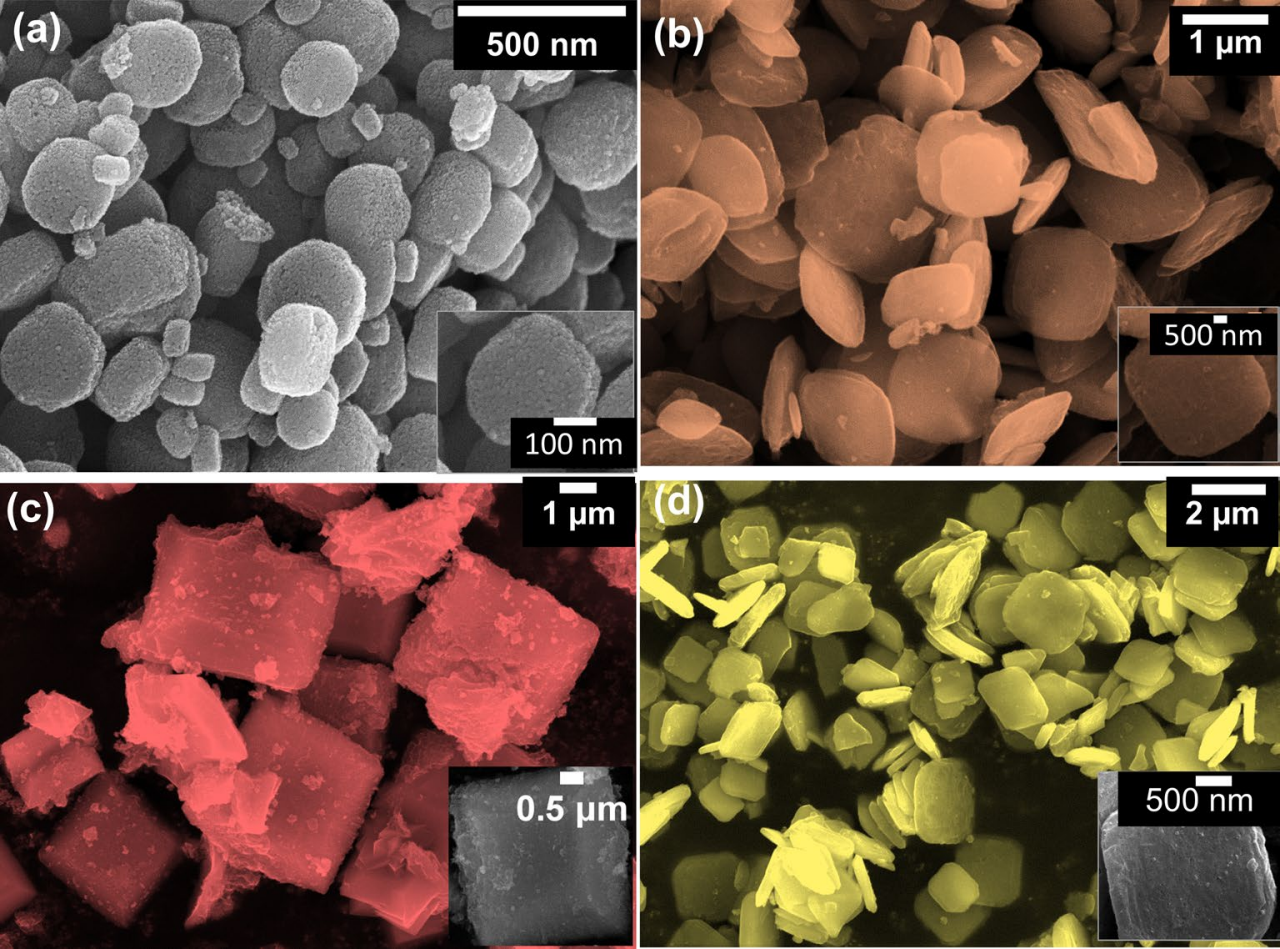
FESEM images of MOF-derived TiO_2_ and 30 mg Mn, Fe and Ni-doped TiO_2_ NCs demonstrating the variation in morphology after dopant inclusion**.**

The thermal stability of the prepared samples was analyzed by the Thermal gravimetric analysis (TGA) and the resultant thermogram were given in Fig. S7. The Field emission scanning electron microscopy (FESEM) images of the pristine and doped sample are presented in Fig. 3. The detailed morphological topographies of the samples before and after TM doping displayed substantial variations both in size and shape. The undoped sample (Fig. 3a) displayed disc-like shapes of dissimilar size (100–400 nm). The surface of the samples was found to be very rough with large pores formed by the interconnected nano-crystallites. Figure 3b–d depicts the 30 wt% Mn, Fe and Ni-doped TiO_2_ samples respectively. After the doping, the average size of all the samples was found to be increased to 1–3 µm. Upon manganese doping, the thickness of nano-dics were reduced with the formation of square shape structures with smooth surface. The iron doping caused the formation of cuboid morphology and was constructed by many tiny NPs and has a highly porous structure as observed in Fig. 3c. Nickel-doped samples showed thin disc-like morphology of few micrometre size and shows smooth surface.

**Figure 4:**
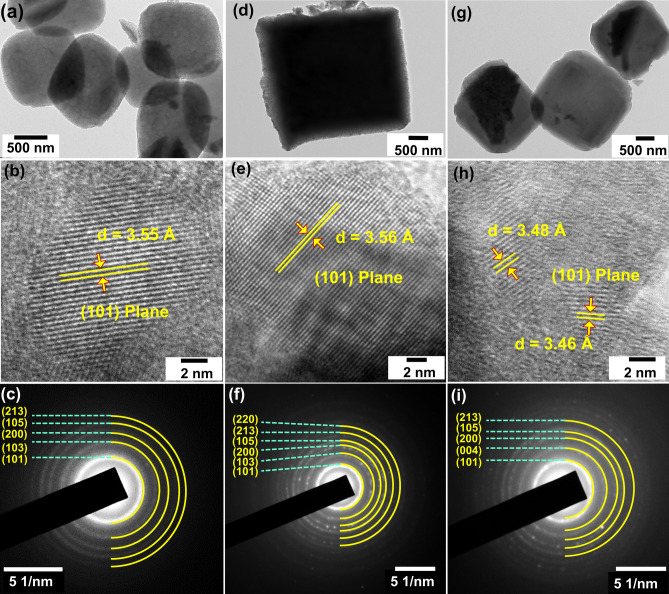
TEM images of MOF-derived 30 mg Mn, Fe and Ni-doped TiO_2_ NCs exhibiting the morphological variations (**a**), (**d**) and (**g**); d-spacing (**b**,**e**,**h**) corresponds to (101) plane of anatase TiO_2_ and SAED pattern (**c**), (**f**) and (**i**).

Further morphological information of the doped samples was understood by High-resolution transmission electron microscopy (HRTEM) analysis presented in Fig. 4. The upper panel (Fig. 4a,d,g) in the TEM image shows the low magnification images of Mn, Fe and Ni-doped TiO_2_ NCs, respectively. The typical morphological distinction can be recognized as the dopant varies. The particles showed an average size of 0.5–2 µm. The high magnification TEM images in the middle panel (Fig. 4b,e,h) confirmed that TiO_2_ NPs have high crystalline properties with the plane spacing of 3.55, 3.56 and 3.48 Å which is in good agreement with (101) plane of anatase TiO_2_. The crystallinity of the prepared nanoparticles was examined by a selected area diffraction (SAED) pattern and prsented in the bottom panel of Fig. 4c,f,i. The bright spots indicate the high crystallinity of doped samples. These small bright spots, made up of rings, substantiate the polycrystalline nature of the TiO_2_ particles. The inter-planar spacing corresponding to diffraction rings is corroborated by anatase TiO_2_ crystal structure obtained from XRD analysis.

**Figure 5:**
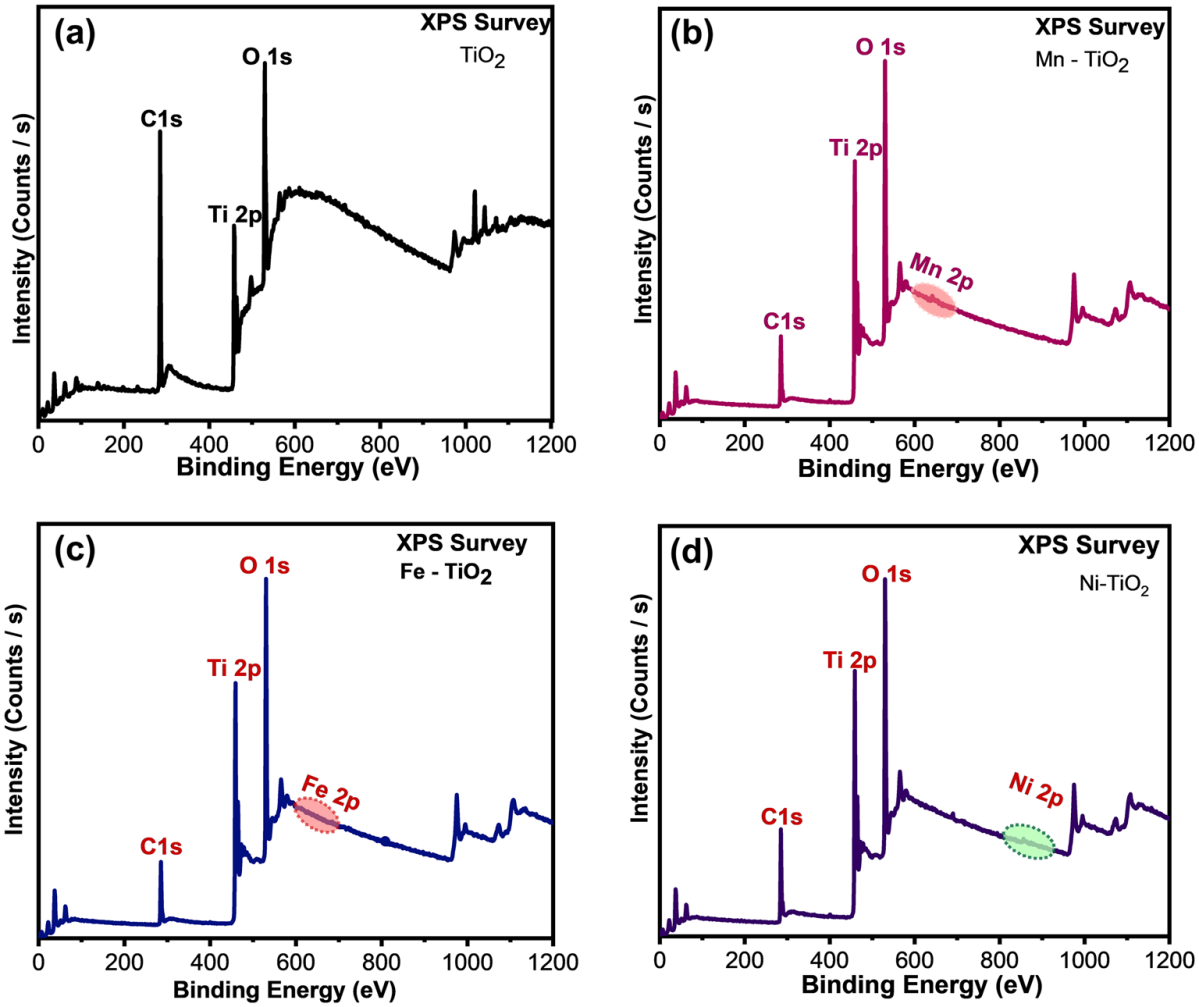
XPS Survey spectrum of (**a**) MOF derived TiO_2_ and (**b**), (**c**) and (**d**) 30 mg Mn, Fe and Ni-doped TiO_2_ NCs, respectively.

**Figure 6:**
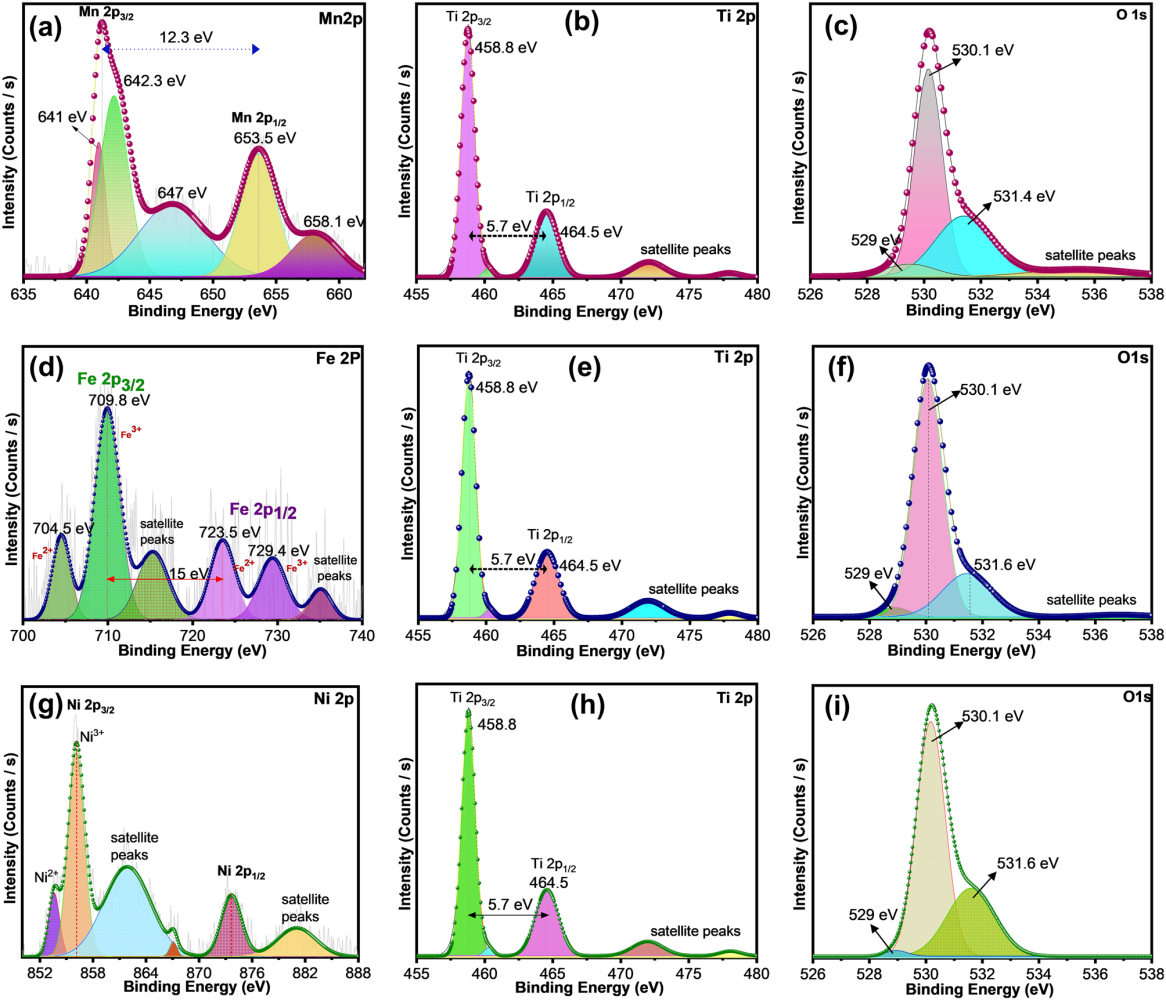
XPS core spectra of MOF-derived 30 mg Mn, Fe and Ni-doped TiO_2_ NCs.

Being surface-sensitive technique X-ray photoelectron spectroscopy (XPS) provides the chemical composition, oxidation number of each element, formation of any secondary phases and the degree of oxygen deficiency of the samples. Figure 5a–d shows the XPS spectrum of pristine TiO_2_ as well as Mn, Fe and Ni-doped TiO_2_, NCs, respectively. The spectra show the existence of C, Ti, Mn, Fe, Ni and O elements in which the appearance of element C is assigned to the carbon-based impurity in the sample. Doped Mn, Fe and Ni elements’ peaks have appeared around 650, 700 and 850 eV, respectively. The core spectra of each element in the doped samples were given in Fig. 6. The core spectra of Mn-doped TiO_2_ exhibited spin–orbit doublets of Mn 2p_3/2_ and Mn 2p_1/2_ with a splitting energy of 12.3 eV (Fig. 6a). The broad and asymmetric peaks indicate the coexistence of dissimilar oxidation states of Mn in TiO_2_ crystal lattice^55^.

The peak fitting suggests that the obtained peaks at around 641, 642.3, and 647 eV are assigned to Mn^2+^, Mn^3+^, and Mn^4+^ species, correspondingly^56^. Additionally, a signal at about 653.12 eV and 658.1 eV can be associated with Mn^3+^ and Mn^4+^, respectively. The intensity of the peaks analogous to Mn 2p is less intense due to the low Mn content in the sample. No characteristic peaks corresponding to the 2p3/2 core level of elemental Mn (637.7 eV) could be observed. The Fe 2p core spectrum (Fig. 6d) demonstrates the existence of two characteristic peaks at 709.8 eV and 723.5 eV, which correspond to the binding energies of Fe 2p_3/2_ and 2p_1/2_, respectively. This revealed that Fe^3+^ is the major charge state of Fe dopant. The deconvoluted peaks of both Fe 2p_3/2_ and 2p_1/2_ can be resolved into multiple peaks. The Fe2p_3/2_ peak has been resolved into three peaks at B.E. positions of 704.5, 709.8 and 715 eV, which correspond to Fe^2+^2p_3/2_, Fe^3+^2p_3/2_, and satellite peaks, respectively. The Fe2p_1/2_ can be deconvoluted into two peaks at binding energies 723 eV and 729.4 eV, which correspond to Fe^2+^2p_1/2_ and Fe^3+^2p_1/2_, respectively. A satellite peak around 735.18 eV was obtained which corresponds to the presence of minor FeO formation in the sample. The Ni 2p high-resolution spectrum shows two peaks at 856 eV and 873.6 eV, respectively which are corresponding to Ni 2p_1/2_ and 2p_3/2_, respectively (Fig. 6d). The main peak at 856 eV is due to the presence of Ni^3+^ whereas the peak at 853.7 corresponds to Ni^2+^. The satellite peaks were observed at 861.8 eV and 880 eV as a result of multiple splitting in nickel, and are typically used for confirming the existence of Ni^2+^. The core electron XPS spectra (Fig. S8a) of pristine Ti 2p display two peaks with a binding energy of 458.8 eV for Ti 2p_3/2_ and 464.5 eV for Ti 2p_1/2_, which are the characteristic binding energy values of Ti with + 4 oxidation state in anatase TiO_2_. It is noteworthy that the XPS spectra of Ti 2p after the doping are found to be same with Ti 2p_3/2_ and Ti 2p_1/2_ binding energies (Fig. 6b,e,h). Other multiple Ti components or chemical shifts were absent, signifying that the doping does not impact considerably the local chemistry of the host Ti atoms in the TiO_2_. Similarly, the O1s core spectra of pristine and doped samples were also not exhibited any extra peaks or shifts in peak position (Fig. S8b, Fig. 6c,f,i). Thus it is confirmed that the anatase crystal structure is not disturbed after the dopant incorporation, which is in good agreement with the literature data^57^.

**Figure 7:**
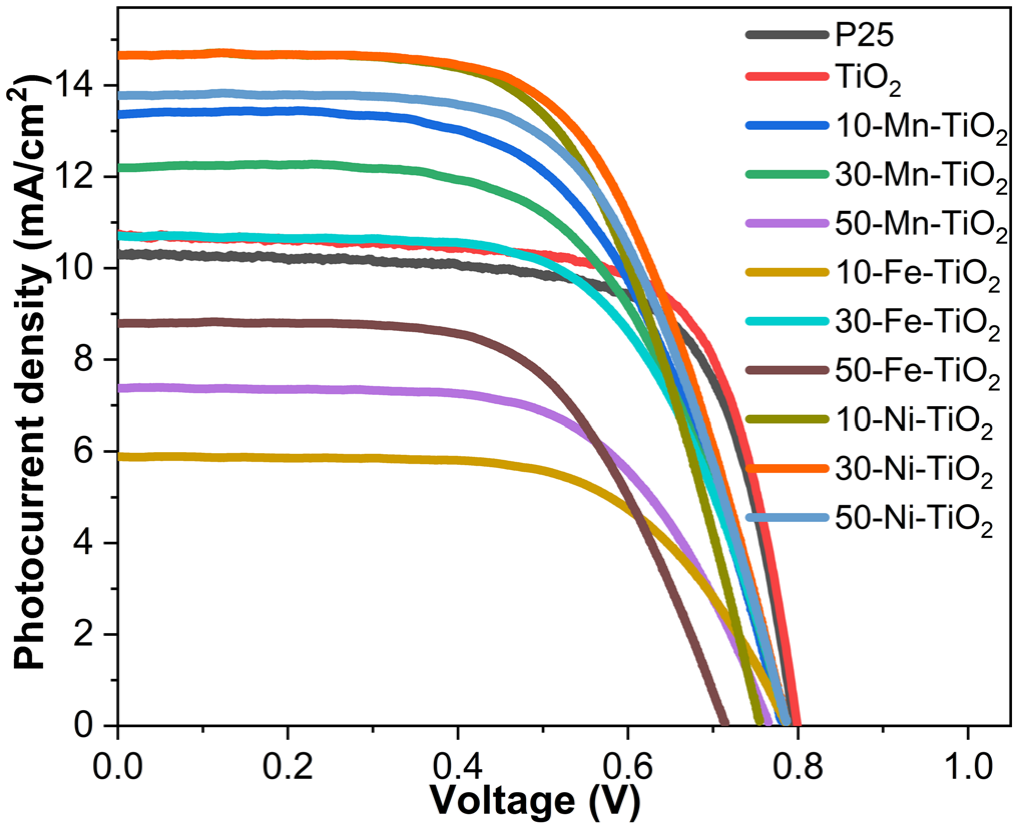
I–V characteristic curve for the fabricated DSSCs with MOF derived TiO_2_ and 10/30/50 mg Mn, Fe and Ni doped TiO_2_ NCs-based photoanodes.

Figure 7 illustrates the J–V curves of the DSSCs fabricated using pristine TiO_2_ and best TM-doped TiO_2_ photoanodes. The detailed photovoltaic parameters extracted from the J–V curves are presented in Table 1.

**Table 1:** Photovoltaic parameters of the fabricated DSSCs with pristine and doped TiO_2_ photoanodes.

Cell	Jsc (mA/cm^2^)	Voc (V)	FF	J_max_ (mA/cm^2^)	V_max_ (V)	PCE (%)
P25	10.31	0.79	70.42	8.91	0.65	5.76
TiO_2_	10.78	0.80	70.31	9.53	0.64	6.06
10-Mn-TiO_2_	13.34	0.78	59.19	11.37	0.54	6.16
30-Mn-TiO_2_	12.23	0.78	59.73	10.54	0.54	5.73
50-Mn-TiO_2_	7.37	0.76	62.43	6.47	0.54	3.52
10-Fe-TiO_2_	5.53	0.79	66.80	5.13	0.57	2.91
30-Fe-TiO_2_	10.69	0.78	63.09	9.33	0.57	5.29
50-Fe-TiO_2_	8.84	0.71	61.19	7.75	0.49	3.83
10-Ni-TiO_2_	14.65	0.75	61.10	12.92	0.52	6.75
30-Ni-TiO_2_	14.72	0.79	60.66	12.92	0.54	7.03
50-Ni-TiO_2_	13.76	0.79	60.99	12.15	0.54	6.61

Moreover, comprehensive DSSC photovoltaic performance data obtained from the photocurrent density–voltage (J–V) curve of five fabricated devices each for various transition metal-doped photoanode materials were given in Table S1. The DSSCs fabricated using the commercially available P25 Degussa photoanode is kept as control which showed a PCE of 5.76% with J_sc_ = 10.31 mA/cm^2^ and Voc = 0.79. The TiO_2_ NCs which were synthesized through MOF route showed enhanced PCE of 6.06% with improved Jsc and Voc of 10.78 mA/cm^2^ and 0.79 V, respectively. This may due to the high surface area of TiO_2_ NCs that was inherited from the parent MOF structure. Further, to improve the PCE, the doped TiO_2_ NCs were used as photoanodes. Different DSSC devices were made with series of TM-doped samples at varying concentration (10, 30 and 50 mg). 10 mg manganese incorporated 10-Mn TiO_2_ sample showed PCE of 6.16%. When dopant concentration was increased to 30 mg, the PCE was decreased to 5.7% with decreased J_sc_ (12.22 mA/cm^2^). Interestingly the Voc was increased to 0.78 V. Further increase in dopant concentration decreased the PCE to 3.52%. The dopant Fe was found to be infelicitous for photoanode applications^58^. Incorporation of nickel in TiO_2_ is found to be very promising. Even for the low concentration of Ni (10 mg), the PCE obtained was 6.75% with J_sc_ of 14.65 mA/cm^2^ and Voc of 0.75 V. A maximum PCE was obtained for DSSCs fabricated using 30 mg nickel-doped TiO_2_-based photoanodes. More increment in nickel concentration (50 mg) has decreased the PCE by 6.61%.

**Figure 8:**
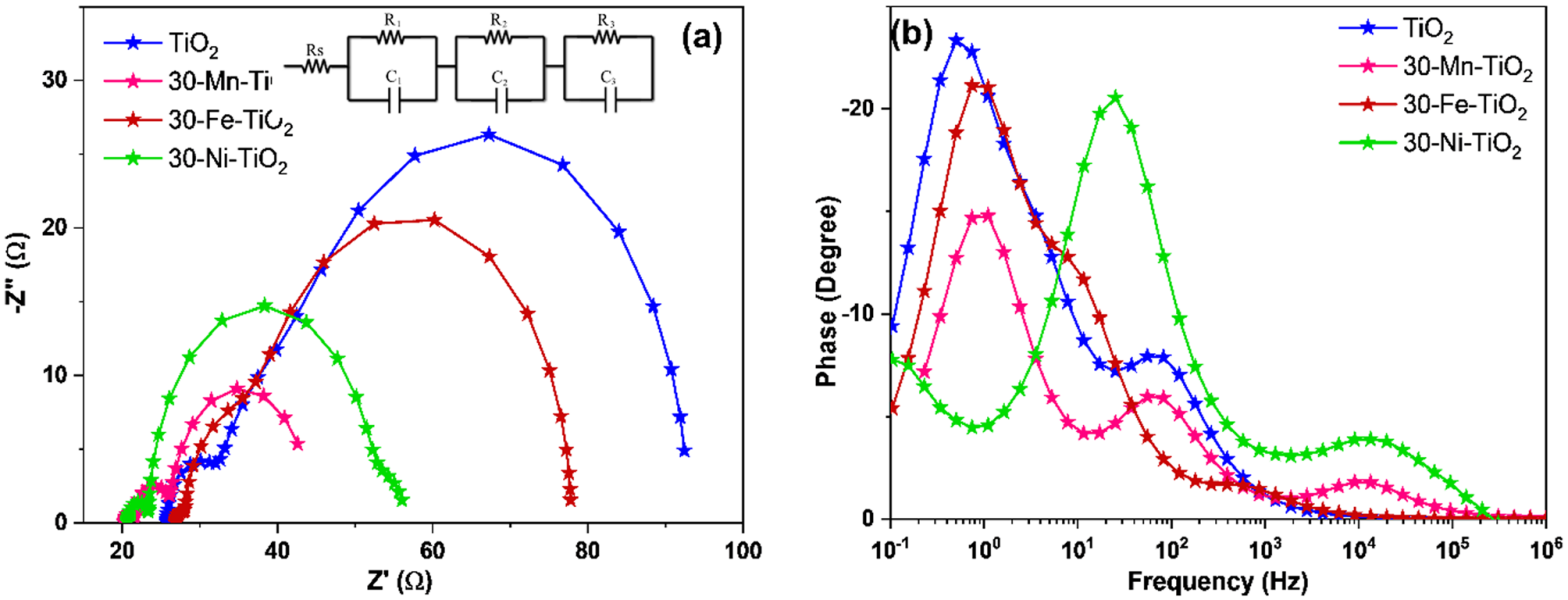
(**a**) Nyquist and (**b**) bode plot of fabricated DSSCs with doped MOF derived TiO_2_ as photoanodes.

To further understand the electron transport and recombinations of photoinjected electrons taking place in DSSCs, electrochemical impedance spectroscopic (EIS) of the fabricated devices were carried out. Figure 8 shows the two EIS plots (Nyquist and bode) of the fabricated DSSCs^59^. Figure 8a represents the Nyquist plot which exhibits three major semicircles, designating the internal interface resistance offered by 3 RC components. Each semicircle, in particular, has a distinct frequency that corresponds to the maximum resistance and specifies the electron lifetime in that specific interface. The constructed DSSCs in open circuit state are considered for the EIS study under 1 sun illumination. The cell parameters are determined by fitting of the obtained graph with the suitable equivalent circuit given as the inset figure in Fig. 8b. It can be noted that the equivalent circuit denotes 4 interfaces present in the DSSCs. The first is the series resistance (Rs) offered by the transparent conducting oxide with the semiconductor oxide layer. The second interface offers the RC components R1 and C1 which obtained at the high frequency region of 100 MHz to 10 kHz. This is responsible for the counter electrode electrolyte interface. The third interface observed at 10 kHz to 100 Hz region, belongs to TiO_2_/dye/electrolyte interface (represented by the RC components R_2_ and C_2_); which directly affects the overall performance of the DSSCs. The interface at low frequency field is due to diffusion in the electrolyte or termed as Warburg impedance. The RC components for the Warburg diffusion is denoted as R_3_ and C_3_. The electron lifetime in the semiconductor is calculated from the frequency maximum obtained from bode plot according to the equation given below.

1 $$\tau = 1/(2\pi {\text{f}}_{\max } )$$

where τ is the electron lifetime, f_max_ is the frequency at the peak maxima of Nyquist or bode plot^60^. The extracted impedance parameters are given in Table 2.

**Table 2:** The extracted impedance parameters of the fabricated DSSCs with different doped MOF derived TiO_2_ as photoanodes.

Cell	R_s_ (Ω)	R_1_ (Ω)	R_2_ (Ω)	R_3_ (Ω)	τ (ms)
TiO_2_	25.45	6.72	51.02	9.48	2.01
30-Mn-TiO_2_	20.31	4.58	17.96	1.32	3.37
30-Fe-TiO_2_	25.88	9.17	40.25	1.28	11.95
30-Ni-TiO_2_	20.63	2.82	28.78	4.12	6.52

From the Table 2, it can be seen that DSSCs fabricated with Mn, Fe and Ni doped TiO_2_ photoanodes have lower charge transfer resistance (R_2_) compared to the undoped TiO_2_ photoanode based DSSCs. Despite the fact that DSSCs made with Mn and Fe doped TiO_2_ have lower PCE than undoped TiO_2_, the observed R_2_ values show an improved interaction between the dopant and the dye molecules. Reduced R_2_ values suggest improved electron transport through the semiconductor oxide (here doped TiO_2_ photoanode)^27,61^. In contrast, the low τ value indicates substantial recombination problems. Thus, the electron injection and recombination processes are competitive.

**Figure 9:**
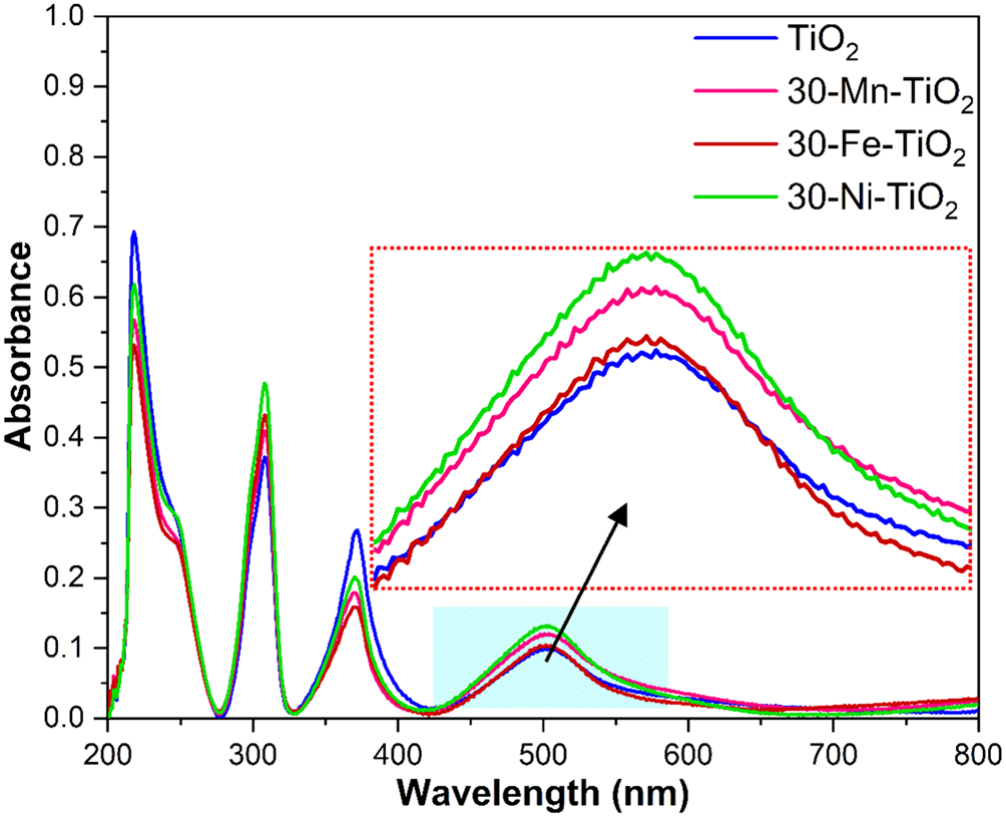
UV–visible absorption spectra of N719 dye desorbed from best-performing different transition metal doped photoanodes through MOF route.

The degree of dye adsorption on photoanodes has a direct impact on the Jsc and hence the PCE of DSSCs. The quantity of dye adsorbed on the photoanode films was estimated by analyzing the UV–visible absorption spectra of solutions containing the dye desorbed from the photoanodes in aqueous NaOH solution. The UV spectra of N719 dye desorbed from various doped photoanode films are shown in Fig. 9. The molar extinction coefficient was used to calculate the amount of dye adsorbed on photoanode layer and the obtained values were 18.34 × 10^–7^ mol cm^−2^, 22.32 × 10^–7^ mol cm^−2^, 19.48 × 10^–7^ mol cm^−2^ and 23.86 × 10^–7^ mol cm^−2^ respectively for TiO_2_ and 30 wt % Mn, Fe and Ni doped TiO_2_ based photoanodes. The findings are consistent with the trend in Jsc values. To compare the dye desorption amount with the surface area of fabricated doped samples, N_2_ adsorption and desorption of samples were done and the obtained values are 40.8, 22.3 and 65.2 m^2^g^−1^ respectively for 30 wt% Mn, Fe and Ni doped TiO_2_ samples. The results are corroborated with the trend in PCE. To assess the device's performance dependability, the long-term stability of fabricated DSSCs was investigated. Table S2 depicts the performance data with reference to J_SC_, V_OC_, FF, and PCE for a 20-day period and the obtained data revealed good stability of the fabricated cells.

## Discussion

We have synthesized Fe, Mn and Ni doped TiO_2_ crystals via the MIL-125 MOF route. The XRD results showed the presence of anatase phase for pristine, Fe and Ni doped samples. In contrast the Mn doping showed mixed phases of anatase and rutile. The quantification of phases resulted in more than 85% anatase and less than 14% rutile phase in the doped sample. The possible mechanism of rutile phase formation is the ‘self-purification’^62,63^. Here the nanoparticles tend to segregate out the Mn atoms towards the surface, causing more Mn atoms at the near-surface than at the interior, which results in the formation of stable secondary phases in the crystal. Any other secondary phases and impurities were not found from the XRD data which confirms the effectiveness of phase pure preparation of doped structures through MOF route. The identified phases are again confirmed through Raman spectra which corroborated to the XRD results. Ni and Fe doped samples showed only anatase Raman vibration modes. In contrast Mn doped sample showed additional rutile Raman modes. Thermal stability data of synthesized samples were analyzed by TGA analysis. Band-gap reduction of TiO_2_ after doping were identified from the Tauc Plot by UV–Vis absorption spectroscopic analysis. Presence of elements their oxidation states were confirmed through EDS and XPS spectroscopic analysis. We have quantified the atomic% of oxygen from the XPS spectra. It is found that the oxygen vacancy is increased in all the doped samples. O1s spectra were fitted with 3 peaks at binding energies 529 eV, 530.1 eV and 531.6 eV are attributed to lattice oxygen, Ti_2_O_3_ and non-lattice oxygen, which confirms that there are no other mixed oxides are formed. The binding energies of lattice oxygen is not shifted after the doping. However, the area varies for different doped samples. Increase in the area of non-lattice oxygen in the doped samples compared to pristine TiO_2_ indicates the formation of oxygen vacancies. The oxygen vacancies followed the order 30-Ni- TiO_2_ > 30-Mn-TiO_2_ > 30-Fe- TiO_2_. The doping process has affected the morphology of the crystals which is identified from the FESEM and TEM analysis. The surface morphology of the nanoscale materials depends on the nucleation and growth of the crystal. In our synthesis procedure, the Mn, Fe and Ni ions were added during the MIL-125 MOF synthesis step. This affects the final crystal formation with varying the growth mechanism. The samples were used for the fabrication of the photoanodes for DSSCs. The elctron transport properties of the cells were identified through EIS spectroscopy. From the obtained results, Fe doped samples showed high electron lifetime values among others. This can be attributed to its structural properties of having small nanoparticle aggregated cuboid morphology with more porous structure which can results in the confinement of electrons in the CB, thus lowering the PCE and also the migration of charge carriers through the oxide layer. This is supported by the high R_2_ value of the sample. Several other factors such as morphology and crystalline features of the photoanodes, diffusion and evaporation of electrolytes etc. can also affect the PCE of the DSSCs^64^. From the PV characterization studies, maximum PCE was obtained for nickel-doped sample with 30 mg Ni^2+^, which was significantly attributed to the synergistic effect of bandgap narrowing, morphological features, increased oxygen vacancies, increased surface roughness and fewer recombination rates after the incorporation of dopant ions as evident from the I-V, impedance and dye desorption studies^54,65,66^. It is significant that the presence of Ni can inhibit the effective photoinduced electron–hole pairs separation by acting as recombination centers. The surface properties of TiO_2_ improved after the Ni doping. The surface roughness of the material is increased as evident from the dye-desorption studies. Moreover, the crystallinity of TiO_2_ was found to be increased which is confirmed from the improved mobility and reduced recombination as evident from the EIS studies. The hierarchical morphology can induce better interconnection, which offers effective utilization of incident light and facilitates fast electron transport. Table S3 compares the performance of our material synthesized with the other reported MOF derived and porous TiO_2_.

## Conclusion

The pristine and transition-metal doped TiO_2_ nanocrystals were prepared by a novel and facile metal–organic framework synthesis route using MIL-125. XRD, Raman, and TEM analysis confirmed the dominant anatase crystal structure formation of all the samples before and after the dopant inclusion. FESEM analysis confirmed the disc-like morphological features for pristine TiO_2_, which became porous nanostructures after the dopant inclusion. This variation highly influences surface properties and hence the PV performance. Additionally, the observed oxygen vacancies and trap states from Raman and XPS spectroscopic studies also contributed to the enhanced PV performance. Higher carrier concentration by transition metal doping provides rapid electron hopping in TiO_2_, which upsurges the electrical conductivity. A significant PCE of ∼7.03% was achieved in 30 mg nickel-doped TiO_2_ photoanodes owing to its high surface area and enhanced interfacial electron transport characteristics. The EIS results of the fabricated cells further confirms the potential reasons for the improved efficiency. It can be summarized that nickel-doped TiO_2_ helps good dye adsorption and fast electron transportation and reduces the recombination for efficient DSSCs. Further improvement in PCE can be achieved by controlling the thickness of the photoanode and introducing the scattering layer, and the study is in progress. This present study is prophesying to open up a new avenue for designing and fabricating novel photoanode architectures with higher PCE for DSSCs. Finally, the demonstrated MOF route synthesis strategy can be implemented to produce various other oxides with controlled morphologies on a large scale for efficient, clean energy conversion and storage device applications.

## Materials and methods

N, N-dimethyl formamide (99.8%) Nickel(II) chloride hexahydrate (99.99%), Manganese(II) chloride tetrahydrate, Iron(II) chloride, Terephthalic acid (98%), Titanium (IV) butoxide (97%), and Anhydrous ethanol (98%) were purchased from Sigma Aldrich, USA. All the reagents were used without further purification, and deionized water was used for the synthesis process. The solvothermal reaction was carried out in a BAOSHISHAN 100 mL autoclave with a PTFE liner.

### Characterization

All pristine and doped TiO_2_ samples were synthesized via MOF route, and phase identification was made by X-ray diffraction (XRD) technique using a Panalytical-Empyrean X-ray diffractometer, Netherlands, with Cu Kα radiation (λ = 1.54 Å) at 45 kV and 40 mA to scan the diffraction angles from 10 to 80°. The phase identification was made using standard patterns reported in the International Centre for Diffraction Data (ICDD) database. TGA analysis was done using a PerkinElmer, Simultaneous Thermal Analyzer (STA) from room temperature to 900 °C at 10 °C/min under N_2_ atmosphere. FESEM and HR-TEM analysis were carried out using Tecnai G2 F20 instrument from FEI, Netherlands operating at 200 kV. The samples were dispersed homogenously in isopropanol by continuous ultrasonication for the TEM study, drop-casted onto the copper grid, and dried overnight before loading into the equipment. Surface area and porosity measurements were carried out on standard adsorption equipment AUTOSORB-1Q-MP-XR, Quantachrome Instruments, USA, using N_2_ gas with 99.995% purity. The diffuse reflectance spectra were examined via a UV–visible spectrophotometer (VarianCary4000) over a wavelength range of 200–800 nm. N719 dye loading quantity of the photoanode was determined by dye desorption studies from photoanode using 10 mM aqueous NaOH solution. The sample was activated under a vacuum at 150 °C for 3 h followed by nitrogen adsorption − desorption analysis at − 196 °C. The surface composition and chemical states were measured with an Omicron Nanotechnology (Oxford Instruments) X-ray photoelectron spectroscope (XPS) equipped with monochromatic Al Kα radiation. The obtained spectra were fitted employing a Gaussian function. The peak correction was done with reference to the standard carbon 1 s peak obtained at 284.67 eV. I–V and EIS analysis of the masked DSSCs were recorded using the CHI660e from CH Instruments under one sun irradiation by PET Photo Emission Tech with SS50AAA solar simulator with AM 1.5G filter. EIS was studied in the range of 1 Hz to 0.1 MHz with the magnitude of the alternating current (AC) 10 mV and the obtained results were fitted using Z-fit software.

### Synthesis

#### Synthesis of TiO_2_ and doped TiO_2_ via MOF route

The synthesis procedure is reported in our previous publication, and the detailed procedure is as follows^41^. Ti-based metal–organic frameworks (MIL-125) were synthesized through a simple solvothermal method. To a mixed solution of terephthalic acid (3 g), anhydrous methanol (6 mL), and anhydrous N, N-dimethyl methanamide (54 mL), butyl titanate (1.56 mL) was added slowly with constant stirring for 10 min. The whole mixture is then transferred into a 100 mL Teflon-lined stainless steel autoclave and is allowed to heat at 150 °C for 24 h. After the solvothermal reaction, a white suspension of MOF (MIL-125) was obtained and separated carefully by washing many times with methanol. The sample is then dried in a vacuum oven at 80 °C for 12 h.

The white precipitate obtained after solvothermal reaction (MIL-125) was annealed at 400 °C at a rate of 2 °C min^−1^ for 5 h to get the pure titania powder stored in the sample vial. To synthesize the doped TiO_2_ samples, MnCl_2_·6H_2_O, FeCl_2_·6H_2_O, and NiCl_2_·6H_2_O (10, 30, and 50 mg) were added to the latter reaction mixture, and the same synthesis protocol was followed. Eventhough 50% doping can be considered as composite, we have mentioned it as doping throughout for unification.

### Photoanode preparation

To Prepare DSSC photoanode, the prepared samples (0.5 g) were dispersed into 4 mL ethanol by ultrasonication for 15 min. 1.5 g terpineol and 0.10 g ethyl cellulose were added into the mixture and then sonicated to form a homogenous viscous paste. The prepared paste was deposited on cleaned fluorine-doped tin oxide (FTO) glass substrate (transparent electrode with a typical square sheet resistance of 10 Ω/sq and overall transmittance of about 85% in the visible range) screen-printing method to get a thickness of ~ 13–15 μm with area of 0.25 cm^2^. After drying, the coated substrates were heated in a furnace (450 °C for 2 h). After reaching room temperature, the substrates were immersed in 0.5 mM solution of N719 dye (Sigma-Aldrich) for 12 h.

### Counter electrode preparation

Pt-counter electrodes were prepared by drop-casting one or two drops of 5 mM H_2_PtCl_6_ solution (in isopropanol) onto the FTO and sintering at 400 °C for 20 min. The transparent electrode obtained was used as the counter electrode without further heat treatment or modification.

### Assembling of DSSC

The assembly process of a DSSC involves sandwiching of photoanode and cathode together with surlyn film of 30 μm thickness as a spacer. Between the sandwiched electrodes, 2–3 drops (~ 3-5µL) of redox couple electrolyte consisting of 0.06 M 1-butyl-3-methyl imidazolium iodide, 0.03 M of iodine solution (I_2_), 0.10 M guanidinium thiocyanate, 0.5 M 4-tert-butyl pyridine dissolved in a mixture of acetonitrile and valeronitrile (volume ratio, 85:15), were introduced using a syringe.

## Supplementary Information

Supplementary Information.

## Data Availability

The datasets used and/or analysed during the current study available from the corresponding author on reasonable request.
