# Supplementary material for: Photovoltaic performance of MOF-derived transition metal doped titania-based photoanodes for DSSCs
Source: Sci Rep. 2023 Apr 18;13:6345. doi: 10.1038/s41598-023-33565-6 (PMC10113198; doi:10.1038/s41598-023-33565-6)
Supplement: Supplementary file 1 — Supplementary Information. [file 41598_2023_33565_MOESM1_ESM.docx]

**Electronic Supplementary Information**

**Photovoltaic Performance of MOF-Derived Transition Metal Doped Titania-Based Photoanodes for DSSCs**

C. Nizamudeen^1‡,^R. Krishnapriya ^1,2,‡,^ M.S. Mozumder^3^, and A-H. I. Mourad ^1,4,5*^, T. Ramachandran ^1^

**Note: This supplementary information contains supplementary figures S1-S8 and table S1-S2**


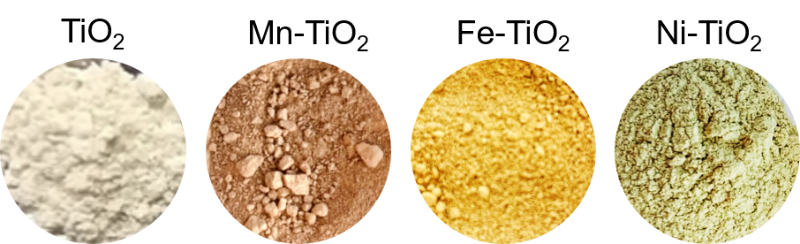


**
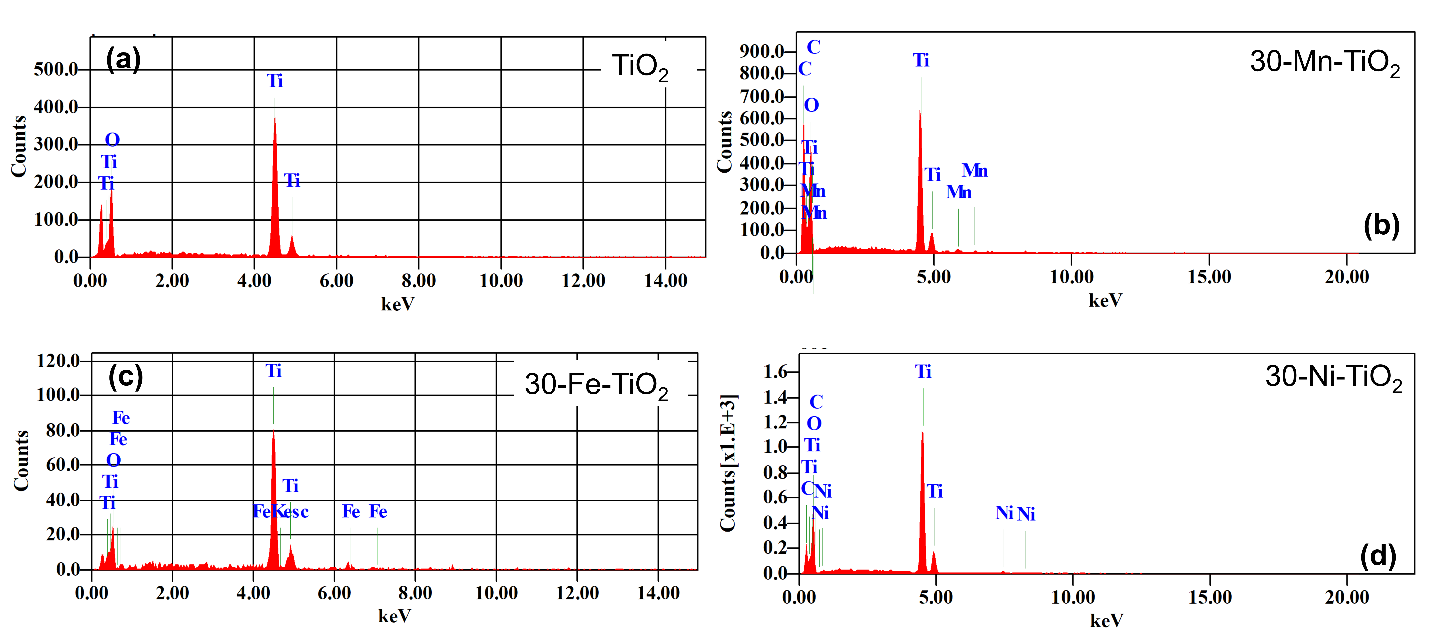
Figure S1**. Photographic images of MOF derived TiO_2_ and doped TiO_2_ samples.

**Figure S2**. EDS spectrum of MOF-derived TiO_2_ and transition metal-doped TiO_2_ samples.


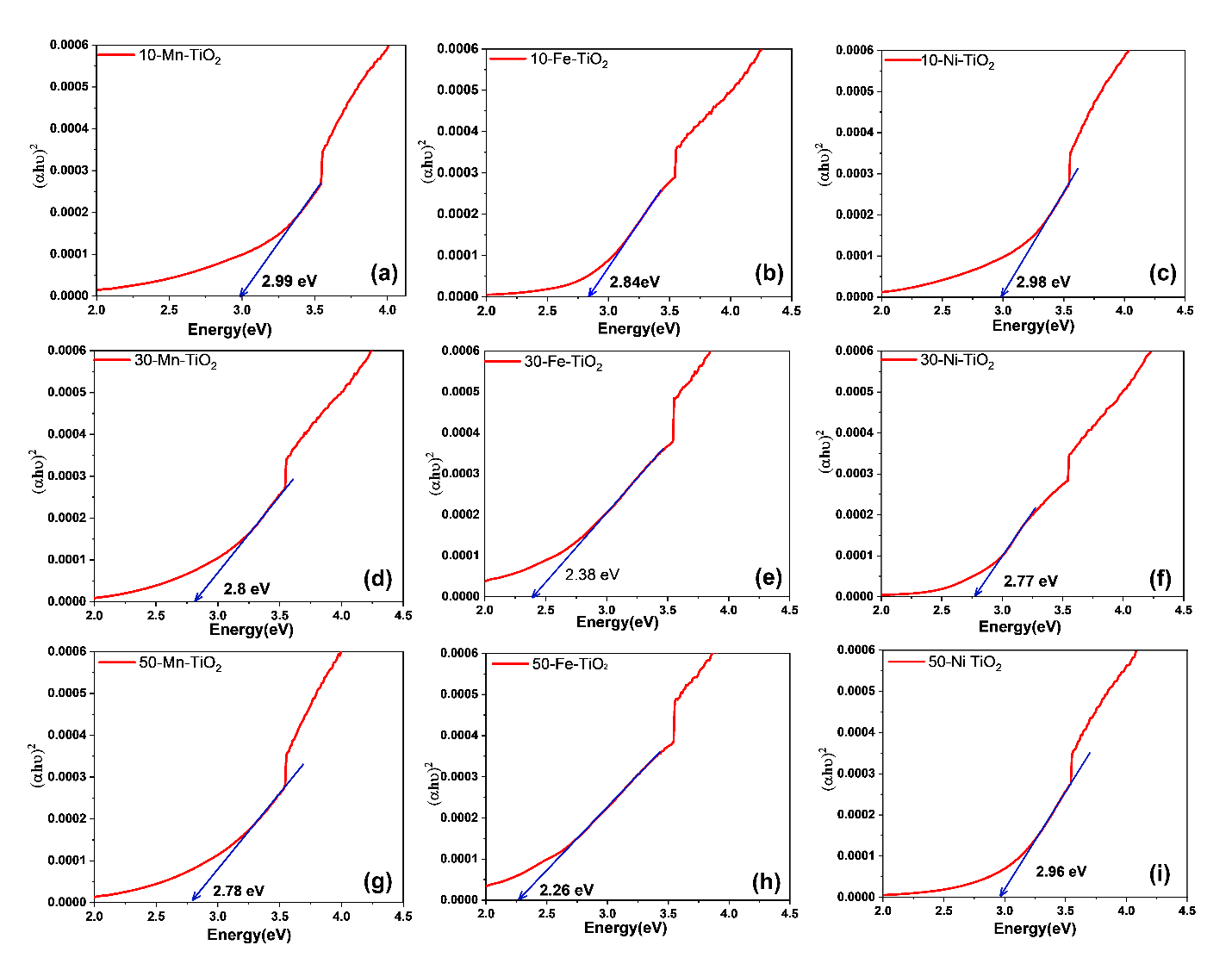


**Figure S3**. Band-gap energy from UV-Vis spectra by Kubelka-Munk plot for pristine and doped samples with 10, 30 and 50 mg of dopants.


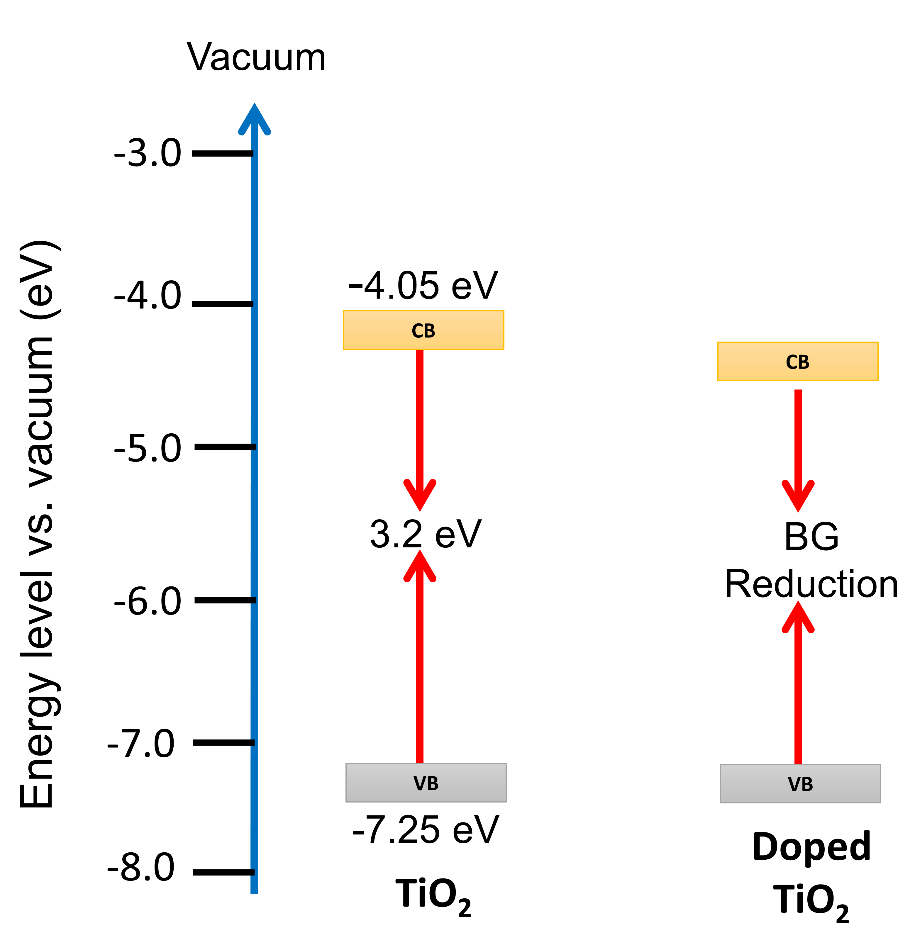


**Figure S4.** Band diagram of TiO_2_ and doped TiO_2_ samples


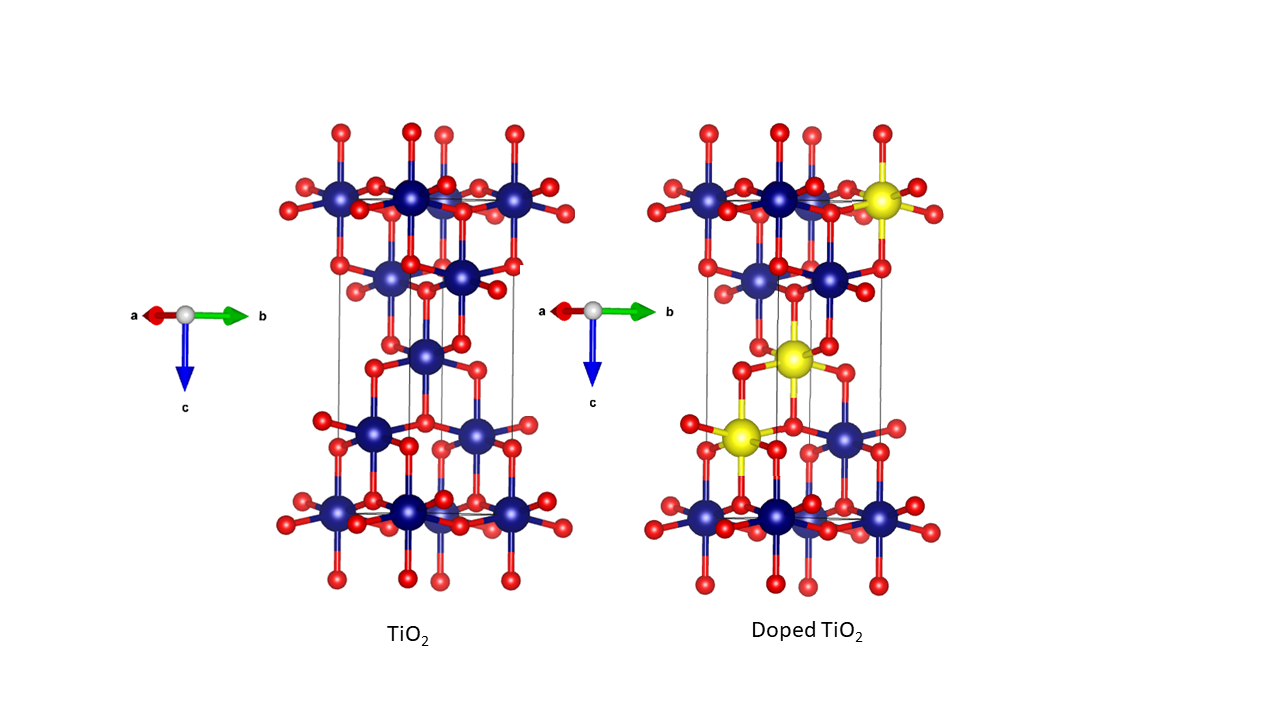


**Figure S5.** Crystal structure of TiO_2_ and doped TiO_2_ samples





**Figure S6**. Raman spectra of Mn doped TiO_2_ showing anatase and rutile mixed phase

**Figure S7**. The TGA curves for pristine and doped samples via MOF route exhibiting high thermal stability.


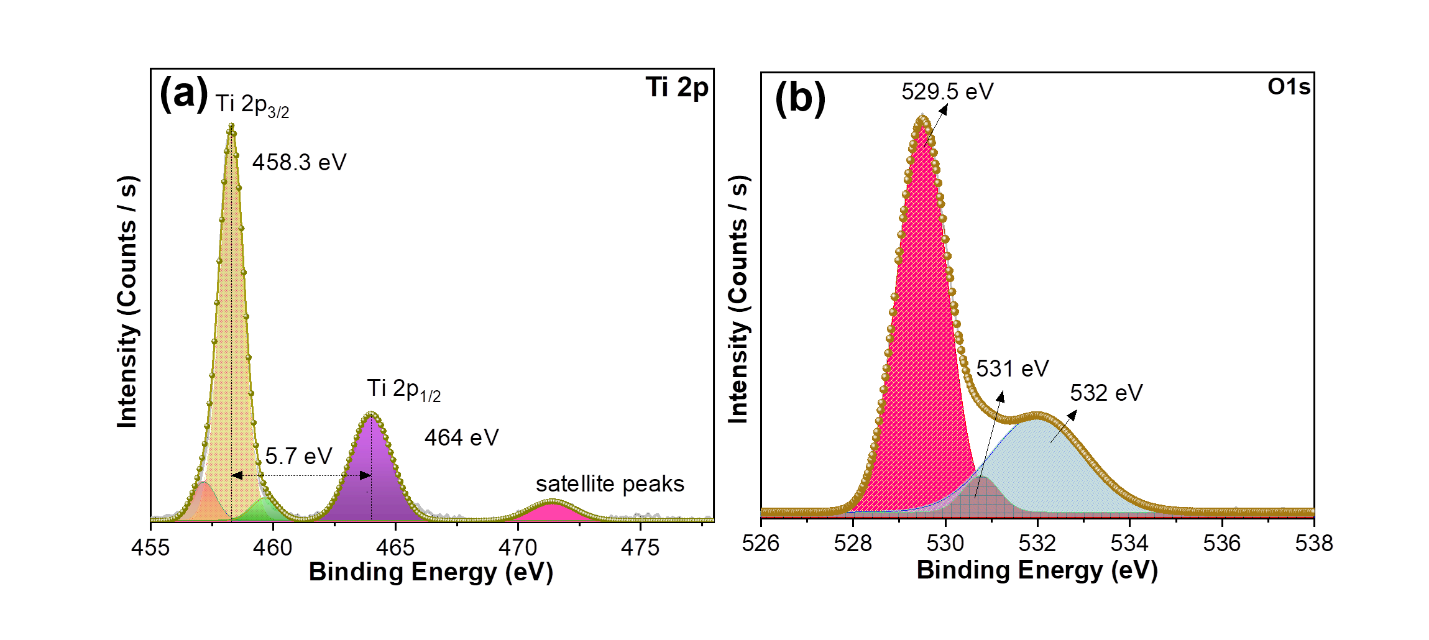


**Figure S8**: XPS core spectra of pristine MOF-derived TiO_2_

| Parameter | Material | Cell 1 | Cell 2 | Cell 3 | Cell 4 | Cell 5 |
| --- | --- | --- | --- | --- | --- | --- |
| J_sc_ | P25 | 10.3237 | 10.3087 | 10.3037 | 10.3137 | 10.3187 |
|  | TiO_2_ | 10.7931 | 10.7751 | 10.7691 | 10.7811 | 10.7871 |
|  | 30 Mn-TiO_2_ | 13.3778 | 13.3538 | 13.3458 | 13.3618 | 13.3698 |
|  | 30 Fe-TiO_2_ | 10.7317 | 10.7167 | 10.7117 | 10.7217 | 10.7267 |
|  | 30 Ni-TiO_2_ | 14.6751 | 14.6541 | 14.6471 | 14.6611 | 14.6681 |
| Voc | P25 | 0.8035 | 0.7885 | 0.7835 | 0.7935 | 0.7985 |
|  | TiO_2_ | 0.8045 | 0.7970 | 0.7945 | 0.7995 | 0.8020 |
|  | 30 Mn-TiO_2_ | 0.7854 | 0.7734 | 0.7694 | 0.7774 | 0.7814 |
|  | 30 Fe-TiO_2_ | 0.7915 | 0.7825 | 0.7795 | 0.7855 | 0.7885 |
|  | 30 Ni-TiO_2_ | 0.7955 | 0.7805 | 0.7755 | 0.7855 | 0.7905 |
| J_max_ | P25 | 8.91769 | 8.90269 | 8.89769 | 8.90769 | 8.91269 |
|  | TiO_2_ | 9.54349 | 9.52249 | 9.51549 | 9.52949 | 9.53649 |
|  | 30 Mn-TiO_2_ | 11.3854 | 11.3644 | 11.3574 | 11.3714 | 11.3784 |
|  | 30 Fe-TiO_2_ | 9.34422 | 9.32922 | 9.32422 | 9.33422 | 9.33922 |
|  | 30 Ni-TiO_2_ | 12.9376 | 12.9166 | 12.9096 | 12.9236 | 12.9306 |
| V_max_ | P25 | 0.6571 | 0.6421 | 0.6373 | 0.6472 | 0.6522 |
|  | TiO_2_ | 0.6423 | 0.6332 | 0.6331 | 0.6361 | 0.6391 |
|  | 30 Mn-TiO_2_ | 0.5514 | 0.5364 | 0.5314 | 0.5414 | 0.5464 |
|  | 30 Fe-TiO_2_ | 0.57251 | 0.56351 | 0.56051 | 0.56651 | 0.56951 |
|  | 30 Ni-TiO_2_ | 0.55402 | 0.53902 | 0.53402 | 0.54402 | 0.54902 |
| FF | P25 | 70.4319 | 70.4169 | 70.4119 | 70.4219 | 70.4269 |
|  | TiO_2_ | 70.3246 | 70.3096 | 70.3046 | 70.3146 | 70.3196 |
|  | 30 Mn-TiO_2_ | 59.2799 | 59.2619 | 59.2559 | 59.2679 | 59.2739 |
|  | 30 Fe-TiO_2_ | 62.7984 | 62.7834 | 62.7784 | 62.7884 | 62.7934 |
|  | 30 Ni-TiO_2_ | 61.0618 | 61.0438 | 61.0378 | 61.0498 | 61.0558 |
| PCE | P25 | 5.77328 | 5.75828 | 5.75328 | 5.76328 | 5.76828 |
|  | TiO_2_ | 6.06876 | 6.05676 | 6.05276 | 6.06076 | 6.06476 |
|  | 30 Mn-TiO_2_ | 6.16843 | 6.15043 | 6.14443 | 6.15643 | 6.16243 |
|  | 30 Fe-TiO_2_ | 5.29797 | 5.28297 | 5.27797 | 5.28797 | 5.29297 |
|  | 30 Ni-TiO_2_ | 7.04368 | 7.02418 | 7.01768 | 7.03068 | 7.03718 |

**Table S1**: A comprehensive DSSC photovoltaic performance data obtained from photocurrent density-voltage (*J–V)* curve of five fabricated devices each for various transition metal-doped photoanode materials

| Cell | PCE | | | | Voc | | | |
| --- | --- | --- | --- | --- | --- | --- | --- | --- |
|  | 0 days | 5 days | 10days | 20days | 0 days | 5 days | 10days | 20days |
| TiO_2_ | 6.0608 | 5.9522 | 5.8752 | 5.7700 | 0.7995 | 0.7971 | 0.7947 | 0.7923 |
| 30-Mn-TiO_2_ | 5.7260 | 5.6235 | 5.5507 | 5.4513 | 0.7841 | 0.7817 | 0.7794 | 0.7771 |
| 30-Fe-TiO_2_ | 5.2879 | 5.1932 | 5.1260 | 5.0342 | 0.7841 | 0.7817 | 0.7794 | 0.7771 |
| 30-Ni-TiO_2_ | 7.0307 | 6.9048 | 6.8154 | 6.6934 | 0.7875 | 0.7851 | 0.7828 | 0.7804 |
|  | Jsc | | | | FF | | | |
| TiO_2_ | 10.7811 | 10.7272 | 10.6736 | 10.5668 | 70.3146 | 69.6115 | 69.2634 | 68.9171 |
| 30-Mn-TiO_2_ | 12.2259 | 12.1648 | 12.1039 | 11.9829 | 59.7307 | 59.1334 | 58.8377 | 58.5435 |
| 30-Fe-TiO_2_ | 10.6880 | 10.6346 | 10.5814 | 10.4756 | 63.0983 | 62.4673 | 62.1550 | 61.8442 |
| 30-Ni-TiO_2_ | 14.7180 | 14.6444 | 14.5712 | 14.4255 | 60.6597 | 60.0531 | 59.7528 | 59.4541 |

**Table S2** Stabilities of the best-performing DSSCs fabricated using MOF-derived doped TiO_2_ studied over 20 days under ambient conditions.

**Table S3** Comparative PV performance of MOF derived and porous TiO2 DSSCs

| Reference | Material | Synthesis method | *V_OC_/*  V | *J_SC_/*  mA cm^−^*^2^* | FF | *η/%* |
| --- | --- | --- | --- | --- | --- | --- |
| Krishnapriya et. al [1] | MIL 125/Co^2+^ TiO_2_ | MOF route, Solvothermal | 0.78 | 13.96 | 0.63 | 6.86 |
| Chi et. al [2] | MIL 125/TiO_2_ | MOF route, Solvothermal | 0.66 | 17.4 | 0.56 | 6.6 |
| Li et. al [3] | TiO_2_/ZIF-8 | MOF route | 0.75 | 10.28 | 0.69 | 5.34 |
| Dou J et. al [4] | MIL 125/TiO_2_ | MOF route, Solvothermal | 0.768 | 13.99 | 0.67 | 7.2 |
| Verjan et. al [5] | Porous TiO_2_ | Solvothermal | 0.73 | 18.59 | 0.47 | 6.43 |
| Wong et. al [6] | NanoporousTiO_2_ | Electron beam PVD | 0.60 | 14.00 | 0.71 | 6.1 |
| Wang et. al [7] | Porous TiO_2_ | Microwave assisted | 0.60 | 15.6 | 0.53 | 5.0 |
| Muzakkar et. al [8] | S-doped TiO_2_ | Wet chemical synthesis | 0.78 | 12.72 | 0.46 | 4.56 |
| Oviedo et. al [9] | Fe-doped TiO_2_ | Solvothermal / microwave | 0.66 | 13.68 | 0.62 | 5.58 |
| Bae et. al [10] | Cu,Co/TiO_2_ | Ultrasonic assisted | 0.68 | 11.42 | 0.64 | 4.31 |
| This work | MIL 125/ (Mn, Fe, Ni), TiO_2,_ | MOF route, Solvothermal | 0.79 | 14.72 | 0.66 | 7.03 |

**Reference**

1. Krishnapriya, R. *et al.* MOF-derived Co2+-doped TiO2 nanoparticles as photoanodes for dye-sensitized solar cells. *Sci. Rep.* **11**, 1–12 (2021).
2. Chi, W. S., Roh, D. K., Lee, C. S. & Kim, J. H. A shape-and morphology-controlled metal organic framework template for high-efficiency solid-state dye-sensitized solar cells. *J. Mater. Chem. A* **3**, 21599–21608 (2015).
3. Li, Y., Pang, A., Wang, C. & Wei, M. Metal–organic frameworks: promising materials for improving the open circuit voltage of dye-sensitized solar cells. *J. Mater. Chem.* **21**, 17259–17264 (2011).
4. Dou, J., Li, Y., Xie, F., Ding, X. & Wei, M. Metal–organic framework derived hierarchical porous anatase TiO2 as a photoanode for dye-sensitized solar cell. *Cryst. Growth Des.* **16**, 121–125 (2016).
5. González-Verjan, V. A. *et al.* Effect of TiO 2 particle and pore size on DSSC efficiency. *Mater. Renew. Sustain. Energy* **9**, 1–8 (2020).
6. Wong, M.-S., Lee, M.-F., Chen, C.-L. & Huang, C.-H. Vapor deposited sculptured nano-porous titania films by glancing angle deposition for efficiency enhancement in dye-sensitized solar cells. *Thin Solid Films* **519**, 1717–1722 (2010).
7. Wang, H.-E. *et al.* Rapid microwave synthesis of porous TiO2 spheres and their applications in dye-sensitized solar cells. *J. Phys. Chem. C* **115**, 10419–10425 (2011).
8. Muzakkar, M. Z. *et al.* Improved photoactivity of TiO2 photoanode of dye-sensitized solar cells by sulfur doping. *J. Phys. Chem. Solids* 111224 (2023).
9. Oviedo, A. M., Thi, H. T., Van, Q. C. & Nguyen, H. H. Physicochemical properties of Fe-doped TiO2 and the application in Dye-sensitized solar cells. *Opt. Mater. (Amst).* **137**, 113587 (2023).
10. Bae, J. *et al.* Efficiency improvement of dye-sensitized solar cells using Cu, Co/TiO2 photoelectrodes doped by applying ultrasonic treatment. *Appl. Surf. Sci.* 156823 (2023).

***************
